# Poor compliance of recurrent stone formers to increase their fluid intake in spite of repeated recommendation

**DOI:** 10.1093/ckj/sfag051

**Published:** 2026-02-23

**Authors:** Lise Bankir, Michel Daudon

**Affiliations:** Centre de Recherche des Cordeliers, INSERM UMRS 1138, Sorbonne Université, Université Paris-Cité, 75006 Paris, France; Centre de Recherche des Cordeliers, CNRS ERL 8228 - Laboratoire de Physiologie Rénale et Tubulopathies, 75006 Paris, France; Service d’Explorations Fonctionnelles, Assistance Publique Hôpitaux de Paris, Hôpital Tenon, 75020 Paris, France; INSERM UMRS 1155, Sorbonne Université, Hôpital Tenon, 75020 Paris, France

**Keywords:** fluid intake, thirst, Tiselius index, urolithiasis, vasopressin

## Abstract

**Background:**

The aim of this study was to evaluate 24-h urine volume (UV), estimated osmolarity (eU_osm_), and Tiselius APCaOx index (TIS) in 181 calcium oxalate stone formers, and the changes achieved in response to the recommendation to reach a UV of at least 2 l per day, during a follow-up of at least 3 years.

**Methods:**

Patients of each sex were *a posteriori* divided into recurrent (R) and non-recurrent (NR) stone formers according to stone events during follow-up. UV, eU_osm_, and TIS were evaluated at baseline (BSL) and End (END). TIS was also determined in first morning urine. Results were compared by two-way ANOVA or chi square.

**Results:**

Mean follow-up was 6.8 years. The 24-h osmolar excretion did not differ between R and NR in either sex, and remained stable between BSL and END. UV was significantly lower in R than NR, both at BSL and END, and eU_osm_ was 100–150 mosm/kg H_2_O higher in R. In both sexes, the increase in UV observed between BSL and END was significantly smaller in R than NR. TIS values were significantly higher in R than NR. They were higher at END than at BSL only in R patients, suggesting a poor compliance of R to drink more. The threshold of TIS for a high risk of crystalluria was found to be 2.45 in both men and women.

**Conclusion:**

In conclusion, the patients who showed recurrence of lithiasis not only had lower baseline UV, but were also less able to increase UV than those who did not recur, in spite of recommendations to drink more. Based on preliminary information in the literature, a clinical trial could be designed to evaluate, in these patients, if a low dose of a selective V2 receptor antagonist, increasing their diuresis (and thirst), would prevent new stones.

## Graphical Abstract

**Graphical Abstract:**
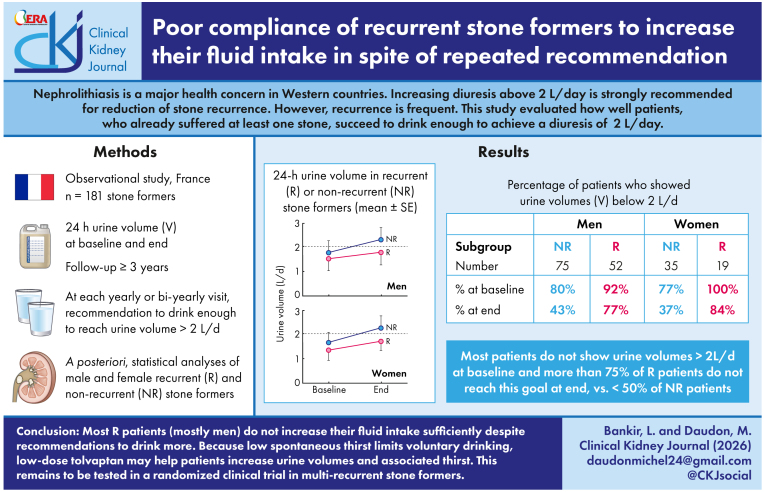

KEY LEARNING POINTS **What was known:**

- Nephrolithiasis affects about 10% of people in Western countries, and prevalence is rising worldwide, resulting in substantial healthcare costs. Calcium oxalate stones comprise 70% of cases. Nephrolithiasis may lead to chronic kidney disease.
- Chronic low fluid intake and inappropriate dietary habits are major risk factors. Increasing diuresis above 2 l/day is strongly recommended for reduction of stone recurrence, but many patients fail to achieve this target.
- The aim of this study was to evaluate how well patients, who already suffered at least one kidney stone, succeed to drink enough to achieve a diuresis of at least 2 l/day, and what proportion of them show stone recurrence(s) during a follow-up of at least 3 years.

**This study adds:**

- In a cohort of 181 stone formers (previously studied for other characteristics), we measured 24 h urine volume (UV), urine osmolarity, and Tiselius index (a marker of lithogenic risk) at baseline and at end of the study after at least a 3 year follow-up. Men and women were studied separately.
- All patients were regularly recommended to increase their diuresis to >2 l/day. Fewer than half of them managed to increase their diuresis above this value. Patients with new stone(s) during follow-up (R) had lower V and increased this V over time less than those with no new stone (NR).
- The baseline Tiselius index of R patients was above 2.45 (a value associated with risk of crystal formation) and below this value in NR patients. These results underline the ongoing difficulty to meet the recommended hydration goals.

**Potential impact:**

- A large number of recurrent stone formers (mostly men) cannot increase fluid intake sufficiently. Recommendations to drink more is not efficacious in these patients. Reducing this major public health risk could be achieved by several other approaches.
- A Tiselius index value above 2.45 in the first morning urine could be useful for predicting a major risk of stone recurrence.
- Because low spontaneous thirst limits voluntary drinking, a pharmacologic approach using a low dose of tolvaptan (drug already used in other diseases) may help patients increase their volume and associated thirst. A randomized clinical trial in multi-recurrent stone formers could evaluate the long-term benefits of this treatment for preventing stone recurrence.

## INTRODUCTION

In Western countries, ∼10% of the population is affected by nephrolithiasis [1–3]. Moreover, kidney stone prevalence is increasing in most countries [4, 5]. Calcium oxalate (CaOx) is the main component of at least 70% of kidney stones [6, 7]. The cost of nephrolithiasis exceeded $2 billion in the early 2000s [8]. Recent reports reveal a link between nephrolithiasis and chronic kidney disease (CKD) [9–11]. In many cases, stone formation is the result of inappropriate dietary habits and low fluid intake [12–15], as highlighted by Zomorodian and Moe [16].

One of the best pieces of advice for efficient stone prevention is to significantly increase water intake with a target above 2 l/day [17]. A high diuresis lowers the concentration of solutes involved in urine crystallization [1, 18–20]. In a previous study [1], we found that crystalluria and stone recurrence were significantly less frequent in patients who showed a urine volume (UV) >2 l/day, while total solute excretion was not significantly lower. Previous reports also provided evidence for a reduced stone recurrence in patients who increased water intake to achieve a diuresis >2 l/day [19, 21]. The main driving force for CaOx crystallization is not the urinary concentration of calcium or oxalate, but the molar product of both concentrations: *p*CaOx = [calcium] × [oxalate]. Thus, doubling the volume of diuresis divides *p*CaOx by four, thus explaining the greater effectiveness of an increased diuresis over any other dietary recommendation [22].

The aim of the present study was to evaluate the adherence of patients who had already experienced a CaOx kidney stone to the recommendation to drink enough to bring their UV >2 l/day. We evaluated daily UV and osmolarity, as well as the Tiselius Ap(CaOx) index risk marker of crystallization [23], at the Beginning and End of a follow-up period of at least 3 years. We compared the changes observed between Beginning and End in male and female patients, and separated, *a posteriori*, those who did not present any new stone nor growth of an *in situ* stone and those who exhibited one or more new stone(s). We also examined the relationship between the AP(CaOX) index and the risk to form crystals in urine, using our laboratory database containing >20 000 first morning urine samples.

## MATERIALS AND METHODS

### Patients

Between January 1984 and December 2000, 651 patients who had formed one or more CaOx stones were investigated repeatedly for blood and 24-h urine chemistry at our clinic, as previously described [1]. These visits were part of their regular care, and thus, no informed consent was required for collecting blood and urine. Moreover, the Declaration of Helsinki, published in 1988, was applied only progressively in France. We first used the data obtained during these visits to analyze retrospectively, in 2005, the relationships between the recurrence of stone episodes and serially determined crystalluria and laboratory parameters in 181 eligible patients [1]. The kidney stone database is registered by the French ‘Commission Nationale de l’Informatique et des Libertés (CNIL)’ with declaration number 1709404 v0.

The present non-interventional study is an extension of this previous study [1] and includes the same 181 patients (127 males and 54 females). The main inclusion criteria were a follow-up of at least 3 years, and available 24-h UV and osmolarity. Additional criteria were a first stone episode at 15 years of age or later, CaOx being the main component of their stone, with <50% calcium phosphate in case of mixed stones. Patients with primary hyperparathyroidism, primary hyperoxaluria, enteric hyperoxaluria, renal tubular acidosis, or systemic diseases were excluded. Any drug therapy that may interfere with calcium metabolism (e.g. thiazides) was withdrawn at least 15 days prior to the baseline evaluation. At each visit (at least once a year), all patients were recommended to ensure a high fluid intake to achieve a daily UV of at least 2 l/d, well distributed over day and evening. During follow-up, radiographic examination (ultra-sound, abdominal X-ray examination) or CT scan was used to detect an increase in size of existing stones or the appearance of new stones. All symptomatic stone episodes were also recorded [1].

All patients had a follow-up of at least 3 years. They were defined as ‘non-recurrent’ (NR) if they had no evidence of a new stone, and ‘recurrent stone formers’ (R) if they suffered at least one new stone during this period. Before the END blood and urine collection, patients were not asked to interrupt drug therapy (if any) that could influence their urine volume. We compared their therapeutic status by analyzing the drug treatments in R and NR patients of both sexes. Thirty four percent of NR patients and 27% of R patients underwent a daily treatment: most of them in both groups with thiazides, and a few with thiazides plus allopurinol or citrate. Analysis by chi-square test revealed no statistical differences between R and NR patients neither between sexes for this therapeutic status.

### Laboratory procedures

At baseline (BSL) and each following visit, a 24-h urine collection was obtained. Patients were given a 3-l container and a 500-ml container for collection of 24-h urine and first morning urine, respectively. Patients were informed of the protocol ensuring proper urine collection [2]. They collected all urine from 8 p.m. (day 1) to 8 p.m. (day 2), and, in another container, the first voided morning urine on the following day (day 3). A blood sample was collected in the morning of day 3. The following measurements were performed: creatinine concentration in plasma; volume of 24-h urine; concentrations in 24-h urine and in first morning urine of creatinine, calcium, phosphate, oxalate, urate, citrate, sulfate, magnesium, sodium, potassium, chloride, and urea.

Estimated urine osmolarity (eUosm, in mosm/l) was estimated according to the formula:


\begin{eqnarray*}
{\mathrm{e}}{{\mathrm{U}}}_{{\mathrm{osm}}} = \ \left( {{\mathrm{Na}} + {\mathrm{K}}} \right)\ \times \ 2 + {\mathrm{urea}}
\end{eqnarray*}

where Na, K, and urea are the concentrations of these solutes in urine, expressed in mmol/l [24].

The Tiselius risk index (TIS) was calculated using two different formulas for 24-h urine (TIS_24-h_) [23], and first morning urine (TIS_fmu_) [25].


\begin{eqnarray*}
{\mathrm{TI}}{{\mathrm{S}}}_{24 - {\mathrm{h}}} = 1.9\ \times {\mathrm{ C}}{{\mathrm{a}}}^{0.84} \times {\mathrm{ Ox }} \times {\mathrm{ M}}{{\mathrm{g}}}^{ - 0.12} \times {\mathrm{ Ci}}{{\mathrm{t}}}^{ - 0.22} \times {\mathrm{ U}}{{\mathrm{V}}}^{ - 1.03}
\end{eqnarray*}

\begin{eqnarray*}
{\mathrm{TI}}{{\mathrm{S}}}_{{\mathrm{fmu}}} = 2.09\ \times {\mathrm{ C}}{{\mathrm{a}}}^{0.84} \times {\mathrm{ Ox }} \times {\mathrm{ M}}{{\mathrm{g}}}^{ - 0.12} \times {\mathrm{ Ci}}{{\mathrm{t}}}^{ - 0.22}
\end{eqnarray*}

Ca, Ox, Mg, and Cit are concentrations of these solutes in urine, in mmol/l, and UV in l.

For many years, we studied in parallel the TIS_fmu_ and the presence of crystals in 20 743 first morning urine samples from several thousand calcium stone formers (12 752 from men and 7991 from women). We determined the value of the TIS above which more than half of the samples contained crystals.

### Statistical analyses

Data are presented as means ± SEM unless otherwise specified. NCSS 2021 software was used. The ‘END’ visit was at least 3 years after the ‘BSL’ visit. Two-way ANOVA was used for comparison between R and NR in both sexes; paired *t*-test to compare BSL and END in the same patients, and chi-square test for categorical variables.

## RESULTS

Demographic and biological data of the 181 patients are shown in Table 1. The mean duration of the follow-up was 6.8 ± 0.3 years. There was no difference between sexes in age at first stone and in duration of follow-up. Men showed significantly higher BMI, 24-h osmolar excretion, and plasma creatinine concentration than women, at both BSL and END. More information about the patients at baseline is provided in our previous study [1].

**Table 1:** Demographic data for the 181 patients subdivided by sex and recurrence.

	Men (*n* = 127)		Women (*n* = 54)		Two-way ANOVA	
	NR	R	NR	R	R vs NR	Sex
*n*	75	52	35	19		
BMI (kg/m^2^)	24.3 ± 0.4	23.8 ± 0.5	22.5 ± 0.6	22.0 ± 0.8	NS	*P* = .002
Age at first stone (y)	35.3 ± 1.5	29.1 ± 0.8*	32.9 ± 2.1	34.1 ± 2.9	NS	NS
Age at Baseline (y)	47.7 ± 1.5	36.7 ± 1.8**	40.1 ± 2.2	41.0 ± 2.7	*P* = .023	NS
Duration of follow-up (y)	6.7 ± 0.5	7.1 ± 0.6	7.4 ± 0.7	6.2 ± 1.0	NS	NS
Plasma creatinine (µmol/l)						
Baseline	94.6 ± 1.5	95.5 ± 1.8	79.2 ± 1.7	80.2 ± 3.0	NS	*P* < .0001
End	97.6 ± 1.6^****^	98.9 ± 1.5***	79.7 ± 1.6	81.7 ± 2.9	NS	*P* < .0001
Urine volume (l/d)						
Baseline	1.74 ± 0.06	1.48 ± 0.07	1.67 ± 0.10	1.26 ± 0.07	*P* < .0001	NS
End	2.26 ± 0.06	1.75 ± 0.07	2.26 ± 0.08	1.59 ± 0.07	*P* < .0001	NS
∆ End minus Baseline	0.52 ± 0.05	0.27 ± 0.06**	0.60 ± 0.08	0.33 ± 0.11**	*P* = .040	NS
Urine osmolarity (mosm/l)						
Baseline	559 ± 22	658 ± 28*	458 ± 28	603 ± 42*	*P* < .0001	*P* = .09
End	433 ± 18	567 ± 23**	350 ± 22	463 ± 32*	*P* < .0001	*P* = .04
Osmolar excretion (mosm/d)						
Baseline	937 ± 33	910 ± 32	728 ± 37	751 ± 54	NS	*P* = .0002
End	948 ± 27	945 ± 46	756 ± 37	742 ± 48	NS	*P* < .0001

Student’s *t*-test for the difference between R and NR in same sex: *P < 0.01; **P < 0001.

Student’s *t*-test for the difference between Baseline and End in NR and R men: ***P = .03; ^****^P = .0075

During follow-up, 40% of men and 35% of women showed a recurrence of urolithiasis. R men were ∼10 years younger at BSL, and showed their first stone 6 years younger than NR (*P* = .00028 and *P* = .008, respectively). No such differences were observed in women. Plasma creatinine did not differ between R and NR patients, neither at BSL nor at END of the study. However, plasma creatinine was significantly increased at END in NR and R men, but unchanged in women.

### Urine data at baseline

**Figure 1:**
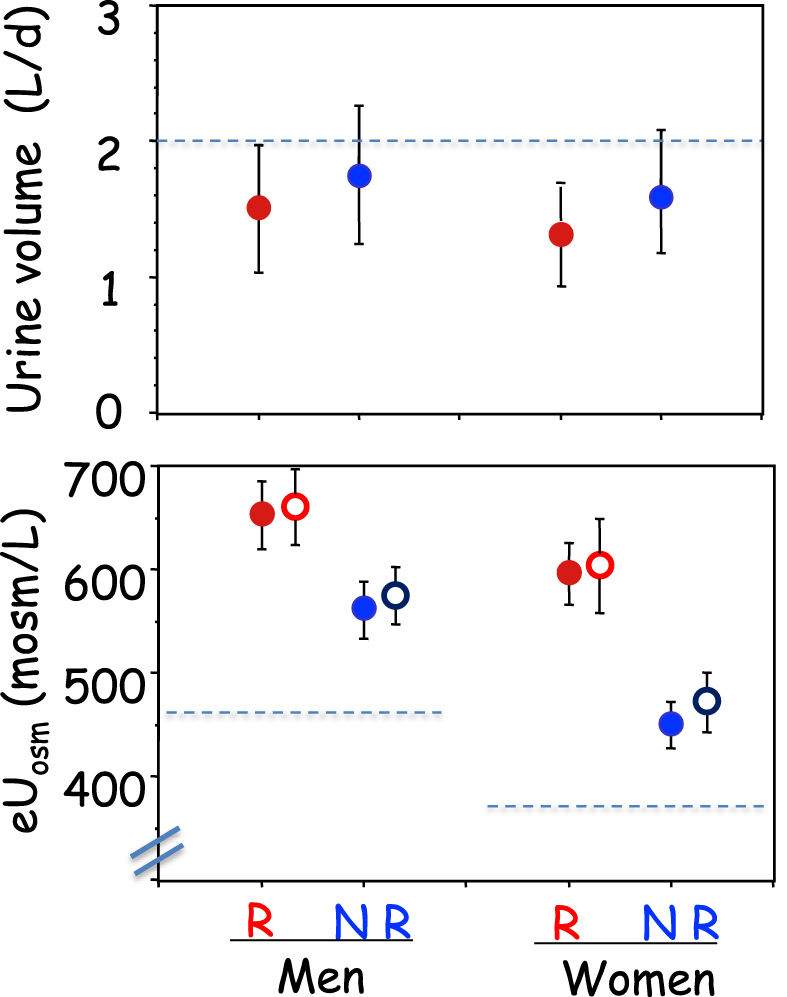
UV and estimated urine osmolarity in 181 patients by sex and recurrence. Mean ± SEM. Red symbols show data at BSL and blue symbols data at the END of the study. Filled symbols show 24-h urine data. Open symbols show data in the early morning urine sample. The horizontal dotted line in the top figure shows a UV of 2 l/day. The horizontal dotted lines in the bottom figure show, for men and women, the osmolarity expected from the total osmolar excretion (shown in Table 1) if UV was 2 l/day. The values are ∼465 and 370 mosm/l, respectively.

Total 24-h osmolar excretion did not differ between R and NR patients in either sex, and remained stable between BSL and END. At BSL, there was no difference in 24-h UV between men and women, but eU_osm_ was ∼15% higher in men than women. However, UV was significantly lower in R than NR in both sexes (two-way ANOVA, *P* = .008) (Fig. 1). Importantly, at BSL, eU_osm_ was significantly higher in R than NR, by 18% in men and 32% in women. eU_osm_ in first voided morning urine at BSL was slightly higher in all four subgroups than in 24-h urine, but this difference did not reach significance.

### Change in urine data during follow-up

**Figure 2:**
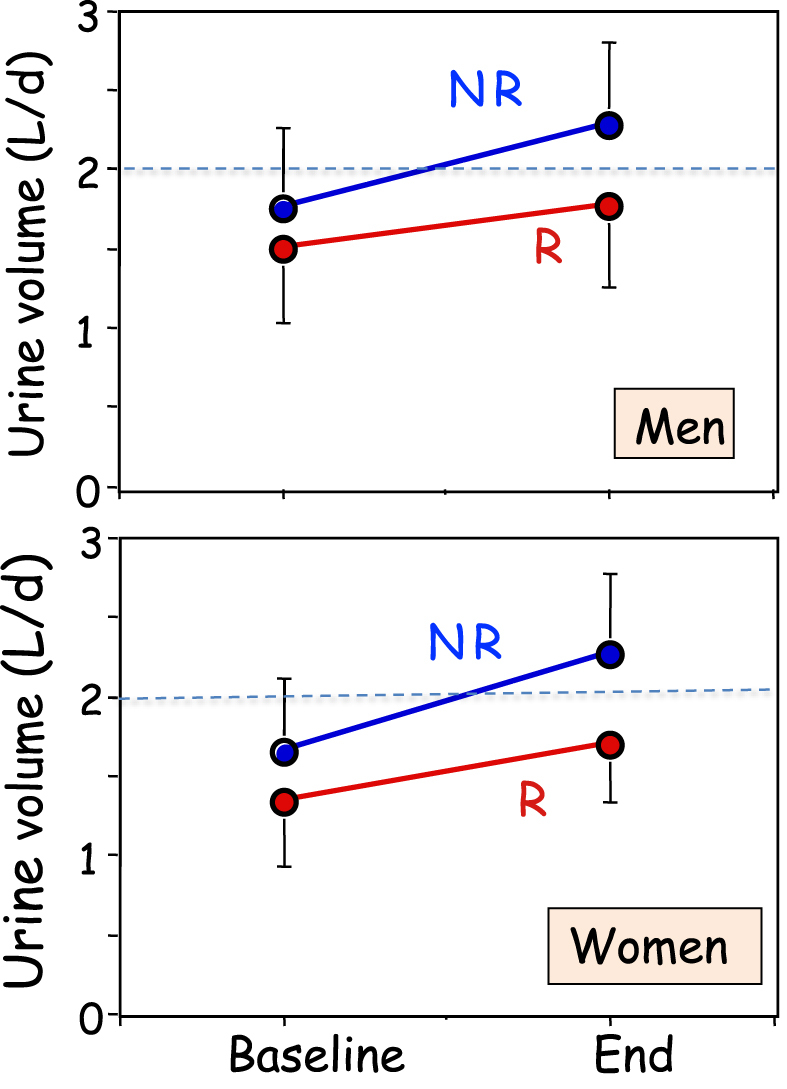
24-h urine volumes in men and women, at baseline and end of the study, in patients who showed (R, red symbols) or did not show (NR, blue symbols) one or more new stones during the study. Means ± SEM.

All patients increased their UV during follow-up (Fig. 2), but R men and women increased UV significantly less than corresponding NR (*P* = .0019). The difference in UV between END and BSL in men and women together amounted to 548 ± 45 ml/d in NR, but only 283 ± 58 ml/d in R (*P* = .00045). Mean 24-h osmolar excretion (mosm/d) remained unchanged in all four groups at END, but eU_osm_ (mosm/l) declined in all groups during follow-up because of the increased diuresis. However at END, eU_osm_ remained significantly higher in R than NR in both sexes (Table 1).

At BSL, the mean daily UV was below the recommended level of 2 l/d in both sexes (dotted line in Fig. 1). Actually, 85% of all patients showed UV below this level (Table 2). This proportion was higher in R than NR of both sexes. At END, 56% still showed UV <2 l/d. And this proportion was much smaller in NR than R patients. Between BSL and END, 37% of NR men and 40% of NR women increased their UV >2 l/d, versus only 15% and 16% in R men and women, respectively (Table 2).

**Table 2:** Number of patients who showed urine volumes below 2 l/d at baseline and end of the study.

	All patients	All patients		Men (*n* = 127)^a^		Women (*n* = 54)^a^	
Group or subgroup (*n*)	181	NR (110)	R (71)	NR (75)	R (52)	NR (35)	R (19)
Number at baseline (%)	154 (85)	87 (79)	67 (94)	60 (80)	48 (92) (b)	27 (77)	19 (100)^b^
Number at end (%)	101 (56)	45 (41)	56 (79)	32 (43)	40 (77) (c)	13 (37)	16 (84)^c^
Chi^2^ (end vs baseline)	***P* = .0001**	***P* = .0001**	***P* = .01**	***P* = .0001**	***P* = .03**	***P* = .001**	NS

a Comparison between men and women at baseline and at end: chi^2^ = NS for both.

b Comparison between NR and R at baseline: chi^2^ = NS in men, and =0.03 in women.

c Comparison between NR and R at end: chi^2^ = 0.001 in men, and =0.001 in women.

### Tiselius index and lithogenic risk

TIS_24-h_, used as a marker of lithogenic risk [25], was significantly higher at BSL in R than NR patients, and decreased significantly less in R at END, in both sexes (Table 3). As expected, TIS was significantly higher in first morning urine than in 24-h urine. At BSL, TIS_fmu_ was >2.0 only in R subgroups and remained >2.0 at END, suggesting a high risk of crystal formation during the night.

**Table 3:** Tiselius index in 24-h urine and in first morning urine in the 181 patients subdivided by sex and recurrence.

	Men (*n* = 127)		Women (*n* = 54)		Two-way ANOVA	
	NR	R	NR	R	R vs NR	Sex
*n*	75	52	35	19		
24-h urine^a^						
Baseline	1.58 ± 0.11	1.83 ± 0.14	1.16 ± 0.14	1.87 ± 0.21	*P* = .012	NS
End	0.89 ± 0.07	1.47 ± 0.09	0.65 ± 0.08	1.36 ± 0.12	*P* < .0001	NS
∆ End minus baseline	−0.69	−0.36	−0.51	−0.51	NS	*P* = .024
Paired *t*-test	*P* < .0001	*P* = 0.0003	*P* = 0.0003	*P* = 0.03		
First morning urine^a^						
Baseline	1.87 ± 0.19	2.66 ± 0.23	1.76 ± 0.32	2.64 ± 0.37	*P* = .01	NS
End	1.45 ± 0.16	2.14 ± 0.21	1.54 ± 0.25	2.61 ± 0.38	*P* = .003	NS
Follow-up	1.57 ± 0.09	2.93 ± 0.14	1.55 ± 0.10	3.10 ± 0.20	*P* < .0001	NS
∆ end minus baseline	−0.38	−0.52	−0.22	+0.08	NS	NS
∆ follow-up minus baseline	−0.30	+0.27	−0.21	+0.46		
Paired *t*-test	*P* = .026	NS	NS	NS		

a The Tiselius index used for first morning urine is different from that used for 24 h urine (see text).

**Figure 3:**
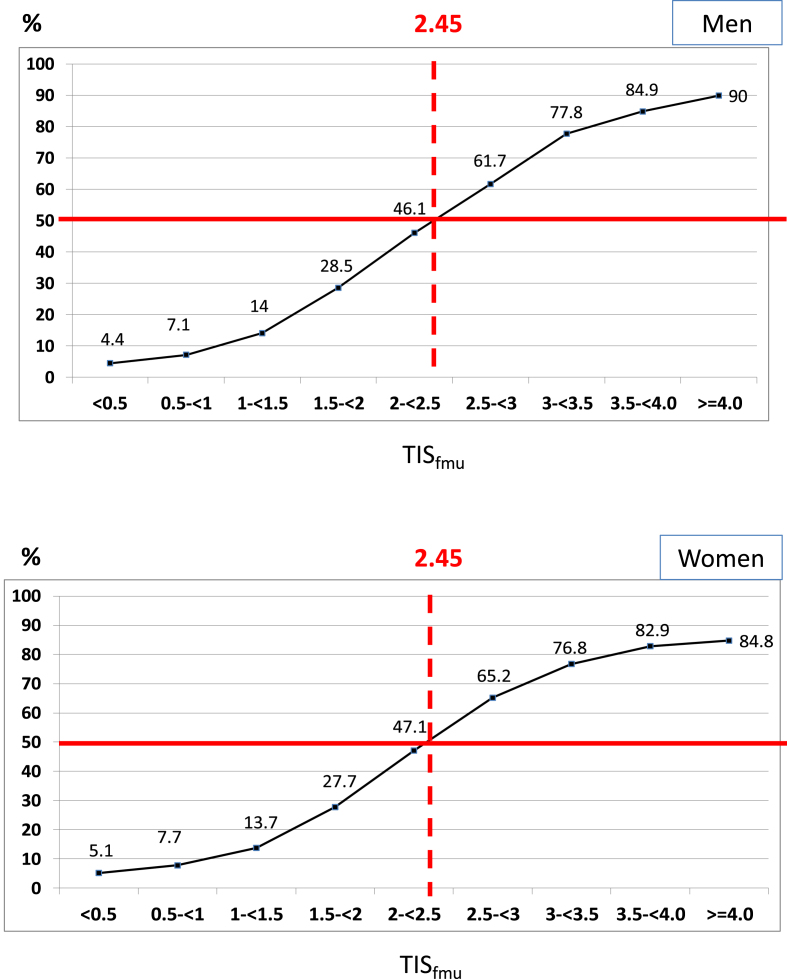
Frequency of crystalluria (%) as a function of TIS_fmu_ value determined in men (12 752 first morning urine samples) and women (7991 first morning urine samples). The threshold value of TIS_fmu_ at which >50% of urine samples contained crystals was found to be 2.45 in both sexes.

As shown in Fig. 3, the threshold of TIS_fmu_ above which more than half of the urines contained crystals was equal to 2.45 in both men and women. At BSL, TIS_fmu_ was above this threshold in R patients and below in NR patients, regardless of sex. During follow-up, it remained above 2.45 in R patients, and below in NR patients.

## DISCUSSION

This retrospective study evaluated the changes in daily UV over at least 3 years in patients who had experienced one or several stone episodes. They were repeatedly recommended to drink enough so as to bring their daily UV to at least 2 l, but only 44% of them reached this goal. Importantly, this study also shows that patients who suffered new stones during follow-up had a lower UV at BSL than those who did not, and increased their UV much less during the study.

Urine osmolarity was already significantly higher at BSL in R than NR. This was not due to a difference in the number of osmoles excreted. Daily UV was not significantly different between men and women. Actually, previous studies reported that the urine output per day is fairly similar in both sexes and the lower osmolar excretion seen in women is associated with a lower urine osmolarity [24, 26, 27]. This is why the recommended UV of 2 l/d was the same for men and women, as already recommended to kidney stone formers in other centers [19].

The TIS_fmu_ did not decrease in R, in spite of a small rise in 24-h UV and a fall in TIS_24h_. This suggests that urine concentration, especially during the night, is an important factor contributing to stone formation. This also suggests that the fluid intake of these patients may not be spread throughout the day in an appropriate way.

Several sex-related differences [28, 29] were again observed here. Osmolar excretion rate and urine osmolarity were higher in men than women [27]. Men who showed a stone recurrence during the study were younger and had undergone their first stone at a younger age than those who did not recur [1, 28], an age difference not observed in women. That stone risks are higher in men than in women is mainly explained by differences in urine biochemistry. Oxalate, phosphate, and uric acid excretions were significantly higher in men than in women (not shown), as already reported by Ferraro *et al*. [22]. These differences in urine biochemistry are partly explained by lifestyle risk factors and dietary habits. Moreover, sexual hormones may explain why women are often less prone to develop calcium oxalate stones than men [29]. However, as shown in Tables 1 and 3, osmolarity and TIS are significantly higher at BSL in R than NR patients, regardless of sex, and remained higher at the end of the study. TIS_24-h_ markedly decreased in NR patients at END in both sexes. In R patients, TIS_24-h_ was only moderately decreased at END and remained close to the threshold proposed for stone recurrence in men (1.47 vs 1.61) and above the threshold (1.36 vs 1.18) in women. Regarding first morning urine, our comparative analysis between the frequency of crystalluria and TIS_fmu_ suggests that the threshold is ∼2.45 in both men and women. TIS_fmu_ decreased below 1.6 in male and female NR patients, but it failed to decrease during follow-up in R patients and remained above the 2.45 threshold value, which may explain stone recurrence.

On average, the difference in UV between END and BSL was much smaller in R than NR patients (*P* < .0001). Assuming there was no major change in extra-renal water losses, this means that R patients did not increase their fluid intake as much as they were recommended to do. This poor compliance may be due to higher vasopressin level and higher thirst threshold in some individuals. Thresholds and slopes for thirst perception and vasopressin secretion are quite variable [30, 31] and are highly heritable [32]. In usual life, urine osmolarity is also quite variable [27], probably reflecting different sensitivity of thirst sensors and vasopressin secretion.

It is difficult to drink more than what thirst commands [33]. In studies involving a voluntary increase in fluid intake, the changes observed were often smaller than what was recommended. In the randomized clinical trial conducted by Clark *et al*. [34], CKD patients were recommended to increase their water intake by 1.0 to 1.5 l above what they were used to drinking daily. In spite of frequent coaching, their UV rose by only 600 ml/d, suggesting that they did not drink enough. In the DRINK study, patients were randomized to prescribed high or *ad libitum* water intakes for 8 weeks. Home monitoring of urine-specific gravity with dispsticks was employed to promote adherence. Only 67% of the participants achieved the urine osmolality target of ≤270 mosmol/kg H_2_O [35]. Frequent instructions plus self-monitoring can improve the adherence to increase water intake [36] for a few months or a year in clinical trials [37], but this cannot last life-long, as would be needed for preventing stone recurrence.

Recurrent episodes of lithiasis put the kidneys at risk of developing CKD [9, 10]. Thus, to prevent stone recurrence, a pharmacological intervention could be proposed to patients who do not succeed to drink enough. The selective vasopressin V2 receptor (V2R) antagonist tolvaptan could most likely help these patients. Tolvaptan increases urine output by reducing water reabsorption in the collecting ducts. As a consequence, thirst is stimulated and the patients increase their fluid intake. The similarities and differences between voluntary increase in hydration and V2R antagonism have been summarized by Bankir *et al*. [38].

Tolvaptan is widely used in patients with hyponatremia [39–41] and to slow the progression of the disease in patients with autosomal dominant polycystic kidney disease (ADPKD) [42–44]. Tolvaptan should allow recurrent stone formers to increase their fluid intake and thus their UV, and reduce their urinary solute concentrations. Actually, two studies showed that the frequency of kidney stones [45] and the lithogenic risk profile [46] were significantly reduced in ADPKD patients treated with tolvaptan, compared to those non-treated. Importantly, the dose of V2R antagonist required to achieve these goals in stone formers should be much lower than that prescribed to ADPKD patients. Thus, the side effects reported in a few ADPKD patients should not be a concern in stone formers.

Cheungpasitporn *et al*. [47], evaluated the influence of one week tolvaptan treatment, 45 mg/day, or placebo in urinary calcium stone formers. Tolvaptan increased UV to 4.8 l/d compared to placebo. It also dramatically decreased urinary super-saturation of CaOx, CaP, and uric acid. A larger and longer randomized clinical trial could be initiated in recurrent stone formers to evaluate the benefits of increasing UV to 2.5 to 3.0 l/d, and lowering urine osmolarity to about 450 mosm/l with a dose of tolvaptan largely lower than the 45 mg/day in Cheungpasitporn’s study. Importantly, the drug should be given with equal doses in the morning and evening to prevent excessive urine concentration during the night.

A strength of this study is that both sexes were considered separately in all analyses. This is important because of the known higher lithiasis risk in men than women. Our study is in agreement with that of Borghi *et al*. [19], showing that recurrent stone formers fail to adequatly increase their urine output. It also has some limitations. First, it is based on only one 24-h urine collection at BSL and at END. However, the relatively high number of patients and the fact that each patient was studied twice should minimize inaccuracies. Second, all patients exhibited idiopathic CaOx urolithiasis. Whether the results can be extrapolated to other forms of urolithiasis requires confirmation.

In conclusion, this study shows that a large fraction of patients who already underwent at least one CaOx stone did not succeed to raise their fluid intake enough to bring their daily UV above 2 l. Based on preliminary information in the literature, for these patients, a pharmacological intervention with a drug, already widely used in other indications, could be really effective. A randomized clinical trial could evaluate the possible benefits of a low dose of tolvaptan in multi-recurrent stone formers.

## Data Availability

The data underlying this article are available in the article and in its online supplementary material.
